# Evaluation of a Recombinant Newcastle Disease Virus Expressing Human IL12 against Human Breast Cancer

**DOI:** 10.1038/s41598-019-50222-z

**Published:** 2019-09-30

**Authors:** Zahiah Mohamed Amin, Muhamad Alhapis Che Ani, Sheau Wei Tan, Swee Keong Yeap, Noorjahan Banu Alitheen, Syed Umar Faruq Syed Najmuddin, Jeevanathan Kalyanasundram, Soon Choy Chan, Abhi Veerakumarasivam, Suet Lin Chia, Khatijah Yusoff

**Affiliations:** 1 Institute of Bioscience, Universiti Putra Malaysia, 43400 UPM Serdang, Selangor Darul Ehsan Malaysia; 2 Department of Microbiology, Faculty of Biotechnology and Biomolecular Sciences, Universiti Putra Malaysia, 43400 UPM Serdang, Selangor Darul Ehsan Malaysia; 3 Xiamen University Malaysia, Jalan Sunsuria, Bandar Sunsuria, 43900 Sepang, Selangor Darul Ehsan Malaysia; 4 School of Foundation Studies, Perdana University, Block B and D1, MAEPS Building, MARDI Complex, Jalan MAEPS Perdana, 43400 Serdang, Selangor Darul Ehsan Malaysia; 5 Department of Biological Sciences, School of Science and Technology, Sunway University, 5, Jalan Universiti, Bandar Sunway, 47500 Subang Jaya, Selangor Darul Ehsan Malaysia; 6 Malaysian Genome Institute, National Institute of Biotechnology Malaysia, Jalan Bangi, 43000 Kajang, Selangor Darul Ehsan Malaysia

**Keywords:** Molecular medicine, Cell vaccines

## Abstract

The Newcastle disease virus (NDV) strain AF2240 is an avian avulavirus that has been demonstrated to possess oncolytic activity against cancer cells. However, to illicit a greater anti-cancer immune response, it is believed that the incorporation of immunostimulatory genes such as IL12 into a recombinant NDV backbone will enhance its oncolytic effect. In this study, a newly developed recombinant NDV that expresses IL12 (rAF-IL12) was tested for its safety, stability and cytotoxicity. The stability of rAF-IL12 was maintained when passaged in specific pathogen free (SPF) chicken eggs from passage 1 to passage 10; with an HA titer of 2^9^. Based on the results obtained from the MTT cytotoxic assay, rAF-IL12 was determined to be safe as it only induced cytotoxic effects against normal chicken cell lines and human breast cancer cells while sparing normal cells. Significant tumor growth inhibition (52%) was observed in the rAF-IL12-treated mice. The *in vivo* safety profile of rAF-IL12 was confirmed through histological observation and viral load titer assay. The concentration and presence of the expressed IL12 was quantified and verified *via* ELISA assay. In summary, rAF-IL12 was proven to be safe, selectively replicating in chicken and cancer cells and was able to maintain its stability throughout several passages; thus enhancing its potential as an anti-breast cancer vaccine.

## Introduction

Newcastle disease virus (NDV) belongs to the family of *Paramyxoviridae*. It has a negative-stranded and non-segmented RNA; with a total viral genome size of 15.9 kb^1^. It has six proteins that include a nucleocapsid protein (NP), phosphoprotein (P), large protein (L), matrix protein (M), hemagglutinin-neuraminidase glycoprotein (HN) and fusion protein (F)^2^. Another two additional proteins, V and W are produced during P gene transcription through RNA editing^3^. NDV strains can be classified into three main pathotypes; velogenic (highly virulent), mesogenic (intermediate virulence) or lentogenic (non-virulent)^4^. NDV strain AF2240 is a Malaysian viscerotropic velogenic strain that was isolated in the 1960s and is currently used in this country as a challenge virus in vaccine trials^3^. The virus has been studied extensively for its oncolytic activity in our laboratory (reviewed in Kalyanasundram *et al*.^5^). In addition, the AF2240 strain has resulted in several successful *in vitro*, *in vivo* and preclinical trials in cancer patients as anticancer agents since 1950s^6–11^.

The development of recombinant NDV has been primarily focused on increasing the oncolytic activity and therapeutic effect. Recombinant NDV expressing several genes such as interleukin 2 (IL2) and interferon-α^12^, granulocyte/macrophage colony-stimulating factor^13^, influenza NS1 protein^14^, tumor necrosis factor, and interferon-γ^15^ have been extensively studied. In this study, we inserted a human IL12 gene into the NDV genome at the position between M and F genes. IL12 is a heterodimeric cytokine that is produced specifically by phagocytic cells as well as antigen-presenting cells and it is known to enhance anti-tumor immune response^16^. In addition, IL12 regulates inflammation through the adaptive and innate immune responses by activating natural killer (NK) and T cells; thus boosting the anti-tumor immune response^17^. A study conducted by Sangro *et al*.^18^ proved that an adenovirus encoding IL12 was able to target liver cancer cells through intratumoral injections. In that study, IL12 was able to induce anti-tumor effects while reducing systemic toxicity^18^.

There are several studies that have investigated the role of IL12 in many types of cancer such as liver, colorectal and pancreatic cancer^18^, renal cancer^19^ and gastrointestinal primary malignancy^20^. The objective of this study was to evaluate the safety, stability and cytotoxic effect of rAF-IL12 against human breast cancer cells *in vitro* and *in vivo* for the potential development of an efficacious anti-breast cancer vaccine.

## Results

### Generation of recombinant NDV (rAF-IL12)

Successful insertion of IL12 gene into the NDV genome was done by cloning the gene between two *Nhe1* restriction sites that were introduced between the M and F genes. The presence of the gene was verified by polymerase chain reaction (PCR) through the detection of size differences in the intergenic region of all NDV genes as shown in Fig. S2. The size of M/F region without IL12 is 377 bp, while in the presence of IL12, the size increases to 2 kb^14^. For further verification of the correct orientation of the IL12 gene, the gene was sequenced and 100% similarity was confirmed.

### Stability and pathogenicity assessment of rAF-IL12

The HA titer of rAF-IL12 was stable when propagated in the embryonated chicken eggs from passage 1 with a starting HA unit of 2^8^ up to passage 10 with a final HA unit of 2^11^ (Table 1). The rAF-IL12 of passages 1, 5 and 10 were used in the subsequent studies to investigate if the stability and pathogenicity of the recombinant virus was maintained throughout the respective passages.

**Table 1:** Viral titers of passaged rAF-IL12.

rAF-IL12 Passage (P)	P1	P2	P3	P4	P5	P6	P7	P8	P9	P10
HA unit	2^8^	2^8^	2^8^	2^8^	2^9^	2^9^	2^9^	2^9^	2^9^	2^11^

Pathogenicity of the rAF-IL12 was determined using the mean death time (MDT) and intracerebral pathogenicity index (ICPI). The rAF-IL12 passages 1, 5, and 10 showed an ICPI of 1.78; indicating towards a velogenic nature (Table 2). However, MDT results indicated towards a more mesogenic nature as compared to that of the velogenic parental AF2240. According to Dortmans *et al*.^21^, although MDT provides useful virulence indication, ICPI is a more accurate measure for the virulence of NDV strains.

**Table 2:** Viral titers and chicken pathogenecity.

Virus	MDT^a^	ICPI^b^
rAF-IL12 Passage 1	68	1.78
rAF-IL12 Passage 5	68.8	
rAF-IL12 Passage 10	68.8	
AF2240	53	

^a^Mean death time (MDT) in eggs measured in hours. Velogenic strains, <60 h; mesogenic strains, 60 to 90 h; lentogenic strains, >90 h.

^b^Pathogenicity of NDV in 1-day-old pathogen-free chicks measured by ICPI. ICPI scale, 0 to 2.0.

### *In vitro* cytotoxicity evaluation of rAF-IL12

**Figure 1:**
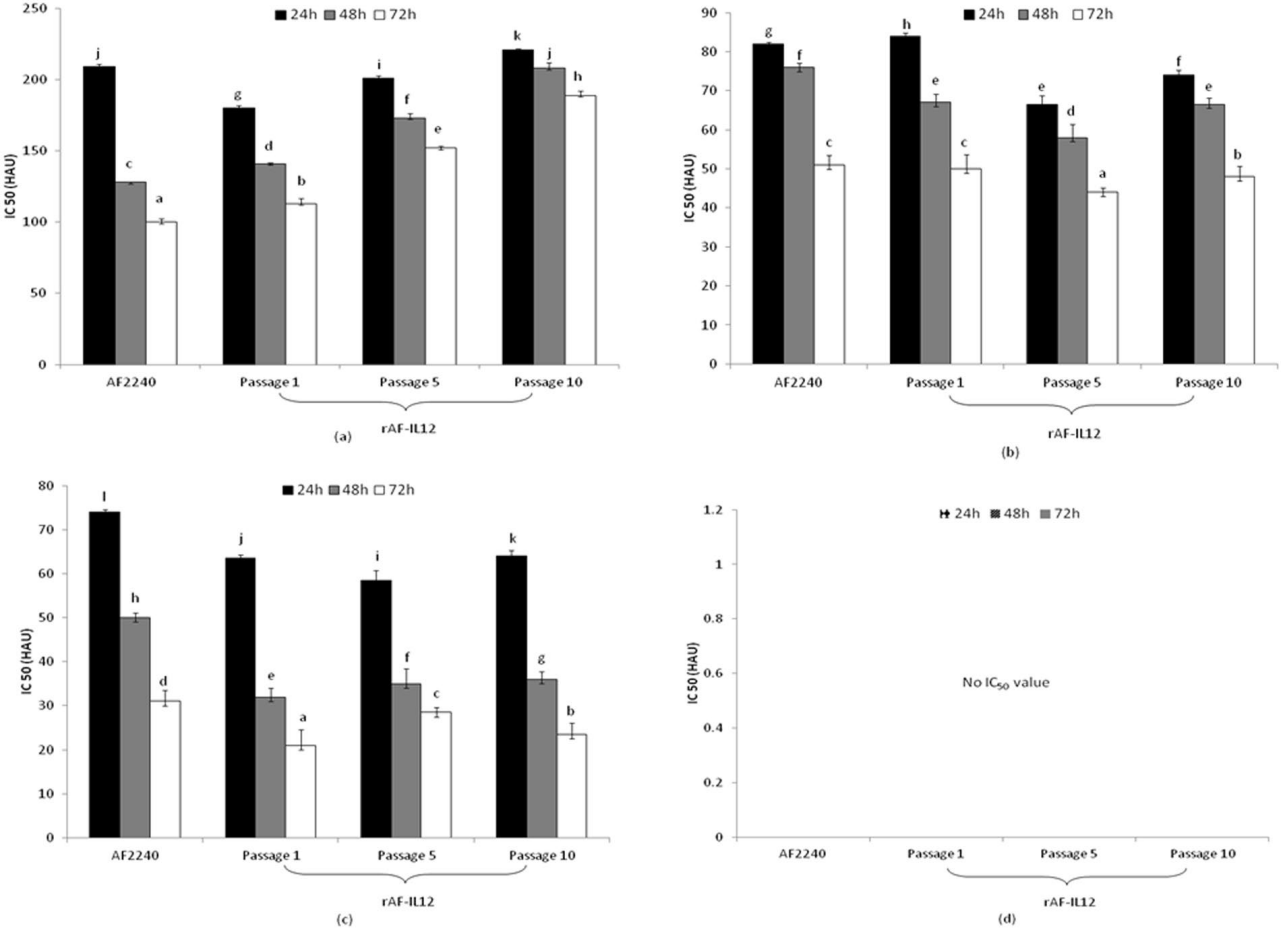
Cytotoxic effects of rAF-IL12 and the parental strain AF2240 against: (**a**) DF-1; (**b**) MDA-MB231; (**c**) MCF-7 and; (**d**) MCF-10A at 24, 48 and 72 h post-inoculation. The values are expressed as the mean values ± standard deviation of triplicate experiments. The mean values as indicated using different superscript letters were significantly different at *p* < 0.05 when compared between parental AF2240- and rAF-IL12 (Passages 1, 5 & 10)-treated groups.

To assess the *in vitro* cytotoxic effect of rAF-IL12 in multiple cell types, MTT assay was conducted using the highest HA unit of each representative virus (as mentioned above). The IC_50_ (half-maximal inhibitory concentration) results in Fig. 1a represents a chicken embryo fibroblast cell line; DF-1, Fig. 1b,c represent two human breast cancer cell lines; MDA-MB231 and MCF-7, respectively and Fig. 1d represents a non-malignant human breast cell line; MCF-10A, when treated with either rAF-IL12 (passages 1, 5 or 10) or the parental NDV AF2240 for 24, 48 and 72 hours. In general, rAF-IL12 from all tested passages were able to significantly (*p* < 0.05) induce cytotoxic effects and inhibit the proliferation in DF-1 and both breast cancer cells. A similar pattern was observed in the parental AF2240-treated groups. Othman *et al*.^22^ demonstrated that NDV AF2240 strain possess cytotoxic activity effects against various types of cancer. However, rAF-IL12 showed enhanced cytotoxic effects against the breast cancer cell lines as compared to AF2240 (Fig. 1a–c); where the rAF-IL12 IC_50_ values were significantly lower (*p* < 0.05).

The MTT results prove that rAF-IL12, at each tested passage was able to significantly induce cytotoxicity against DF-1, MDA-MB231 and MCF-7 and was safe as it caused limited cytotoxic effects against the normal MCF-10A breast cell line.

### Level of IL12 expression by rAF-IL12

**Figure 2:**
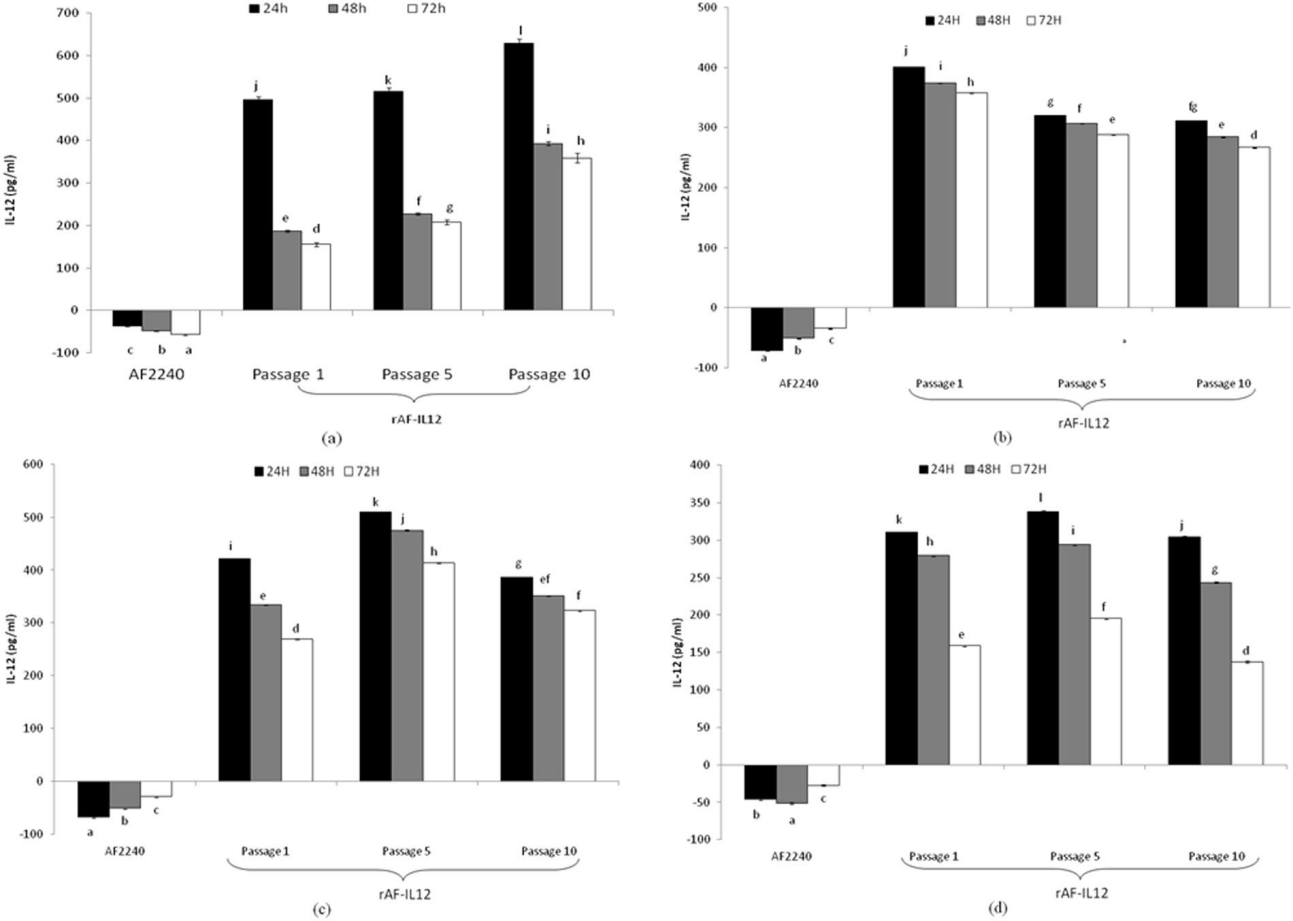
The expression of human IL-12 in multiple cell lines infected with NDV AF2240 and rAF-IL12 (Passages 1, 5 & 10) determined by ELISA assay at 24, 48 and 72 h. (**a**) DF-1 cells treated with NDV AF2240 and rAF-IL12 (Passages 1, 5 & 10), (**b**) MCF-7 cells treated with NDV AF2240 and rAF-IL12 (Passages 1, 5 & 10), (**c**) MDA-MB231 cells treated with NDV AF2240 and rAF-IL12 (Passages 1, 5 & 10), (**d**) MCF10A cells treated with NDV AF2240 and rAF-IL12 (Passages 1, 5 & 10). The values are expressed as the mean values ± standard deviation of triplicate experiments. The mean values as indicated using different superscript letters were significantly different at *p* < 0.05 when compared between parental AF2240- and rAF-IL12 (Passages 1, 5 & 10)-treated groups.

The concentration of IL12 that was secreted by rAF-IL12 passages 1, 5 and 10 in DF-1, MDA-MB231, MCF-7 and MCF-10A cells was measured by an ELISA assay that contained capture antibodies that only recognized the IL-12 heterodimer and not the individual subunits. As demonstrated in Fig. 2, high levels of IL12 were detected in all the rAF-IL12-treated cells.

As compared to the parental AF2240, the concentration of IL12 was significantly (*p* < 0.05) higher in all the tested passages of rAF-IL12 across all tested time points; where the level of IL12 expression was found to be as high as 600 pg/ml at 24 hours post-infection but the expression levels gradually decreased over time. Although there was a significant reduction of IL12 expression in the later time points, the levels were still significantly above 100 pg/ml.

### rAF-IL12 significantly inhibits the growth of 4T1 cells *in vivo*

**Figure 3:**
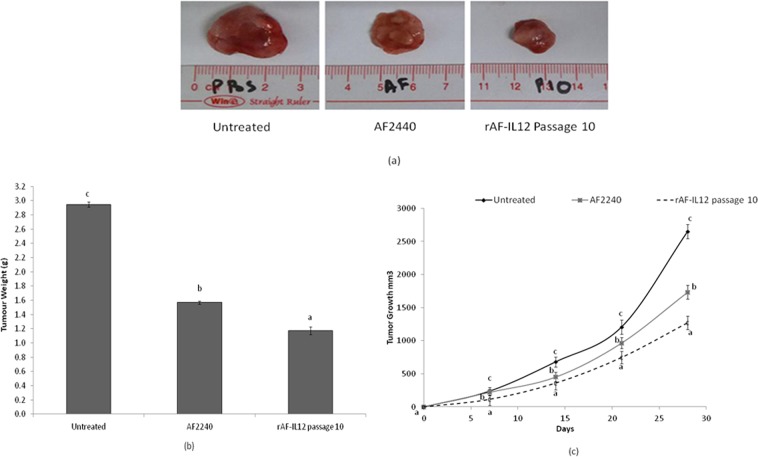
The tumors harvested from untreated, NDV AF2240 and rAF-IL12 passage 10-treated mice (2^7^ HAU/kg) mice. (**a**) Representative images, (**b**) average weight and (**c**) volume of the tumors harvested from untreated, AF2240 and rAF-IL12 passage 10-treated mice. Each value represents the mean values ± S.E.M. There were 9 mice per group. The values are expressed as the mean values ± standard deviation of triplicate experiments. The mean values as indicated using different superscript letters were significantly different at *p* < 0.05 when compared between parental AF2240- and rAF-IL12 (Passages 1, 5 & 10)-treated groups.

In this study, 27 Balb/c mice were subcutaneously injected with 4T1 mouse breast cancer cells to develop tumors. The mice were randomly divided into 3 intra-tumoral treatment groups of 9 mice each. The mice in the first group were treated with rAF-IL12 passage 10 (2^7^ HAU), the second group received an equivalent dose of AF2240 and the third group was treated with PBS. The rAF-IL12 demonstrated greater anti-tumor effects as compared to the parental AF2240 (Fig. 3). Tumor size in rAF-IL12-treated mice was significantly reduced as compared to the AF2240 and PBS-treated mice (Fig. 3a). As shown in Fig. 3b, the relative tumor weight after 28 days of treatment was less in rAF-IL12-treated mice as compared to AF2240-treated mice. The rAF-IL12 treatment resulted in 52% growth inhibition, while AF2240 caused 34.5% growth inhibition as compared to the PBS control group. Thus the tumor burden of rAF-IL12 treated group was significantly reduced (*p* < 0.05) in comparison to that of the AF2240 and control group (Fig. 3c).

### rAF-IL12 reduced mitosis and significantly augments IL12 cytokine expression

**Figure 4:**
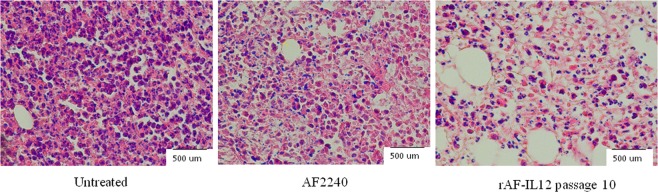
Histological staining (haematoxylin and eosin) of tumors from untreated, NDV AF2240- and rAF-IL12 passage 10-treated mice groups. Condensed round cells stained dark purple represent cells undergoing mitoses. Magnification: X40.

Histological examination of the tumors from the control group identified a high proportion of cancer cells that were mitotic, as demonstrated by the presence of condensed dark purple nuclei that were stained by hematoxylin (indicated by the black arrows) in Fig. 4. However, the number of mitotic cells in the tumors harvested from rAF-IL12-treated mice was reduced as compared to the control group.

**Figure 5:**
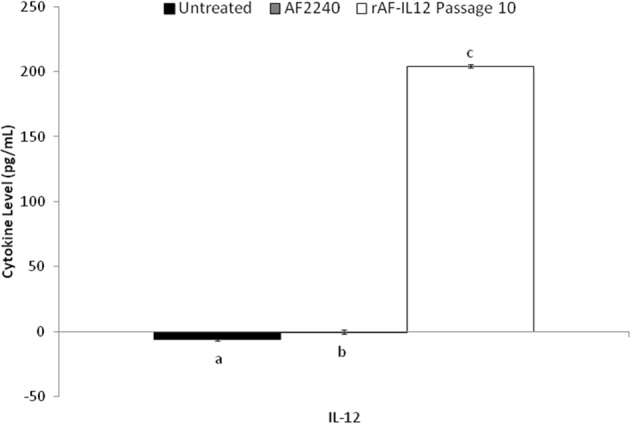
ELISA-based detection of IL-12 levels in the serum of the control (untreated), NDV AF2240- and rAF-IL12 passage 10-treated mice groups. Each value represents the mean values ± S.E.M for n = 9 mice per group. The values are expressed as the mean values ± standard deviation of triplicate experiments. The mean values as indicated using different superscript letters were significantly different at *p* < 0.05 when compared between parental AF2240- and rAF-IL12 (Passages 1, 5 & 10)-treated groups.

The human IL-12 levels were measured in the serum of the rAF-IL12-treated, AF2240-treated and control mice groups. As demonstrated in Fig. 5, the level of IL12 increased significantly (*p* < 0.05) in the rAF-IL12 passage 10-treated mice as compared to both the AF2240-treated and untreated mice. The level of IL-12 was increased up to 204.07 ± 0.66 pg/mL in the rAF-IL12 passage 10-treated mice.

### Quantification of rAF-IL12 in tumor and organs of treated mice

**Figure 6:**
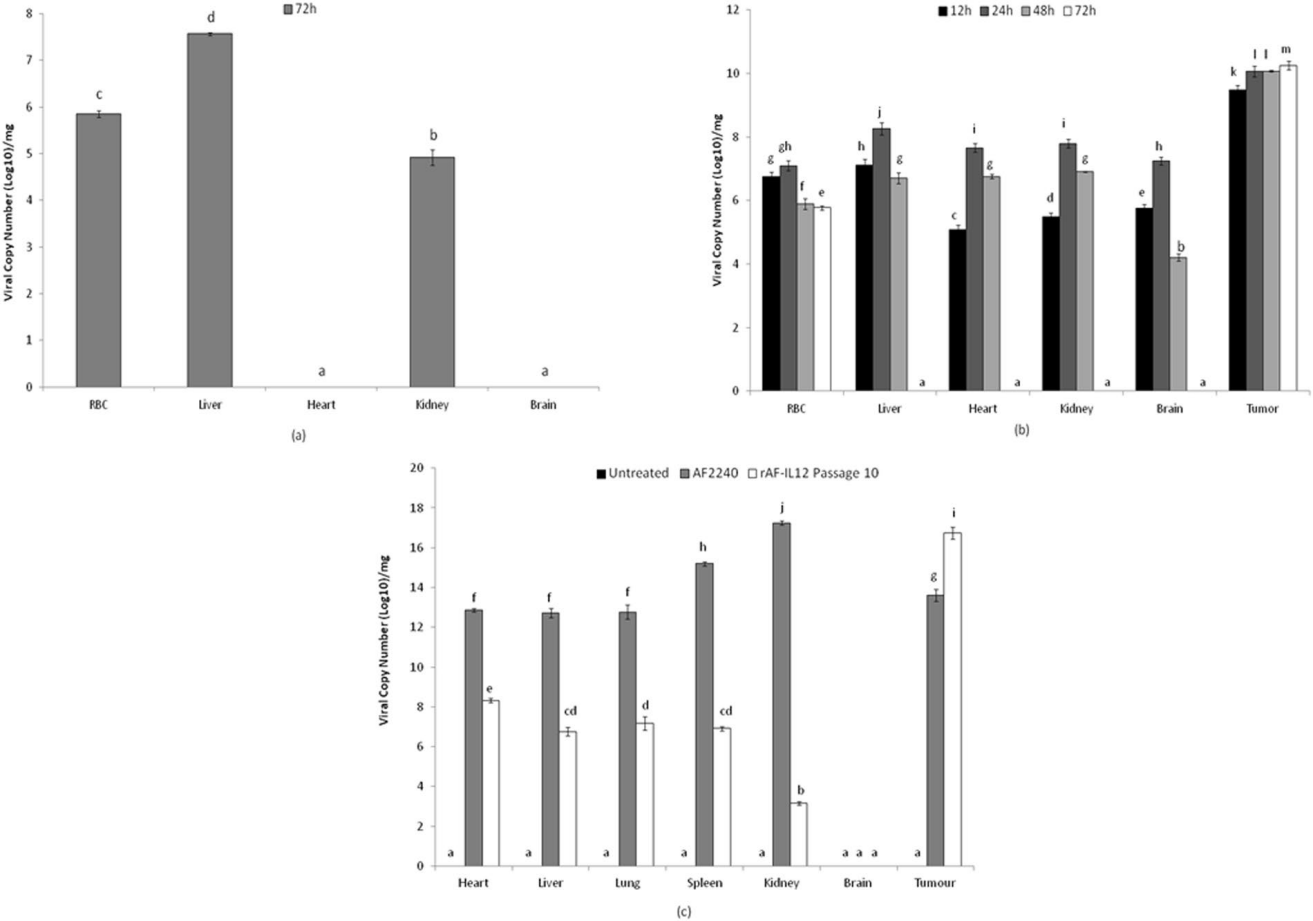
Detection of viral copy number in organs and tumor of treated mice using Taqman quantitative real time PCR. Images represent (**a**) normal (no tumor) and (**b**) rAF-IL12 treated mice after 3 days using Taqman quantitative real time PCR, c) detection of viral copy number in organs and tumor in NDV rAF-IL12 passage 10- and AF2240-treated mice after 28 days using Taqman quantitative real time PCR. Each value represents the mean values ± S.E.M for n = 9 mice per group. RBC = Red blood cells. The values are expressed as the mean values ± standard deviation of triplicate experiments. The mean values as indicated using different superscript letters were significantly different at *p* < 0.05 when compared between parental AF2240- and rAF-IL12 (Passages 1, 5 & 10)-treated groups.

The amount of virus that were detected in the organs and tumors of normal (Fig. 6a) and rAF-IL12-treated mice (Fig. 6b) after 72 hours and the amount of detectable virus detected in rAF-IL12- or AF2240-treated mice after 28 days are demonstrated in Fig. 6c. Interestingly, the virus was present at very low amounts in the red blood cells, liver and kidney, while no virus was detected in the heart and brain after 72 hours in the normal mice. In addition, based on Fig. 6b, there was a significant (*p* < 0.05) decrease in the amount of virus between 12 hours to 72 hours in the organs of the treated mice. The rAF-IL12 was not detected in the organs of the treated mice after 72 hours. However, the amount of rAF-IL12 significantly (*p* < 0.05) increased in the tumors of the mice across time. Across all time points, viral copy number of rAF-IL12 was highest in the tumor. Based on Fig. 6c, there was a significant (*p* < 0.05) decrease in the amount of rAF-IL12 in the treated organs as compared to AF2240. In addition, both viruses were not detected in the brains of the treated mice after 28 days. However, the viral load was substantially increased in the rAFIL12-treated tumors as compared to the AF2240-treated tumors.

## Discussion

The recombinant Newcastle disease virus (rNDV) is selectively pathogenic; causing limited harm to humans^8^. Preclinical studies have been conducted using NDV due to its oncotherapeutic potential; making it a promising non-mainstream oncovirotherapeutic agent^8^. The virus has been found to be a highly effective oncolytic agent with high selectively against cancer cells^23^. A previous study identified NDV as an efficient vector to express and deliver foreign genes for vaccination and gene therapy^1^. Increasingly, the oncotherapeutic potential of using recombinant NDV to carry various different types of genes are being investigated^24^.

In the present study, the IL12 gene was inserted in between the M and F genes to ensure sufficient IL12 expression. Our results show that the IL12 gene was correctly inserted into the genome of AF2240 between the M and F genes. IL12 (P70) was chosen as the fusion gene candidate due to the heterodimeric cytokine consisting of both light chain p53 and heavy chain p40, IL12A and IL12B, respectively. This was done to avoid competitive suppression of IL12 if the IL12 P40-40 homodimer were used^25^. To date, there are no reports of using genetically engineered human IL12 expressing-NDV against breast cancer. Human IL12 has been reported to enhance anti-tumor immune response by selectively stimulating human T cells as a powerful agent against tumors^16,17,26,27^. In this study, the safety, stability and cytotoxic effects of a recombinant NDV, rAF-IL12 was investigated.

Inserting a foreign gene between viral genes can cause changes in the abundance of the mRNAs of the flanking gene^28^. Hence, it is important to identify the optimum site for the foreign gene to be inserted into the genome of NDV so as to ensure efficient levels of expression of the inserted gene and minimize the impact on NDV replication features. Zhao and Peeters^27^ conducted a study to test the differences in expression level of a reporter gene encoding human secreted alkaline phosphatase (SEAP) gene when inserted at different sites of the NDV genome. Based on their study, the expression of the SEAP gene that was inserted between NP and P genes was relatively lower as compared to the insertion between the M and F gene when recovered from embryonated eggs at 4 days. The ability of rAF-IL12 to express IL12 in DF-1, MCF-7, MDA-MB231 and MCF-10A was analyzed in this study. High levels of IL12 were detected upon rAF-IL12 treatment across all the tested cell lines. The stability of IL12 gene expression in rAF-IL12 was also confirmed over 10 passages.

The pathogenicity of the recombinant virus based on MDT and ICPI results was also confirmed. The HA, MDT and ICPI results of rAF-IL12 remained stable and unchanged for each tested passage. Thus, the rAF-IL12 was genetically and pathogenically stable during extensive passages *in vivo*. Replication and pathogenic features of rAF-IL12 was not distinguishable from that of the parent NDV AF2240 strain, proving that the insertion of IL12 gene in the genome did not result in any significant differences in replication^1^. NDV AF2240 has been reported to be cytotoxic against breast cancer^29^. Nevertheless, the effect of rAF-IL12 against breast cancer *in vitro* has not been reported as yet. The recombinant virus was further tested in several cell lines, including the chicken embryonic fibroblast DF-1 cell line, two human breast cancer cell lines; human breast carcinoma cell line MCF-7 and human breast adenocarcinoma cell line MDA-MB231 and a non-malignant human breast cell line MCF-10A by using MTT cytotoxic assay. The MTT results showed that rAF-IL12 was comparable to the parental AF2240 strain; inducing cell death in a dose-dependent manner. The MTT cytotoxic assay also revealed that rAF-IL12 selectively induced cytotoxic effects (IC_50_) in both chicken and human breast cancer cell lines without significant toxicity against the normal MCF10A cell line, which makes it an ideal oncotherapeutic^30^. Our results were similar to a study conducted by Ahmad *et al*.^29^, where NDV AF2240 was tested against two normal cell lines; human umbilical vein endothelial cells and normal fibroblast of human breast cell. Based on their MTT assay results, NDV AF2240 did not induce cytotoxic activity against both normal cell lines. In the present study, similar results were obtained as both rAF-IL12 (passages 1, 5 and 10) and parental NDV AF2240 displayed minimal cytotoxic effect against non-malignant MCF-10A breast cell line; whereby the IC_50_ values were unobtainable.

As compared to NDV AF2240, rAF-IL12 managed to reduce the size and weight of the tumor with higher efficacy. In fact, rAF-IL12 significantly reduced the amount of mitotic figures and number of dividing cells in the tumor tissue as compared to the untreated group. The results suggest that using AF2240 in combination with IL12 expression resulted in a greater anti-tumor effect than using AF2240 alone. A study conducted by Rodriguez-Madoz *et al*.^31^ proved the safety of IL12 when expressed by a semliki forest virus (SFV-enhIL-12) to combat against chronic viral hepatitis and hepatocellular carcinoma in woodchucks. In that study, tumor regressed significantly while inducing the cytokine-related immune response against the viral hepatitis and hepatocellular carcinoma without any clinical signs of toxicity in the woodchucks when treated with the SFV-enhIL-12. In this study, the safety of rAF-IL12 was proven based on the viral load titer results of liver, kidney, lung, spleen, heart and brain of the treated mice models. The quantification of rAF-IL12 based on the viral load titer showed a significant (*p* < 0.05) decrease in the vital organs of the treated mice in comparison to the AF2240 treatment. The detectable viral copy number in the organs after 28 days of treatment (Fig. 6c) can be explained by the twice a week viral treatment. Mice sacrificed on Day 28 of treatment would have been injected with the virus on Day 26; only 48 hours before sacrifice. As demonstrated in Fig. 6b, viral clearance only occurs after 72 hours. In both viral treatment groups, no virus was detected in the brain. In another study, IFN-γ was found to be able to significantly decrease viral load in brain tissues^32^. The secretion of IFN-γ in the brain is activated by IL12; resulting in enhanced immune effector functions for viral clearance^32^. We postulate that a similar IL12-induced IFN-γ-mediated increase of viral clearance occurred in the other vital organs; including the heart of the rAFIL12-treated mice. However, the exact mechanism(s) by which viral clearance is mediated in these organs is yet to be elucidated.

Intra-tumoral viral treatment enhances viral spreading to the tumor site; causing natural killer cells and cytotoxic T lymphocytes directed against viral antigens in the infected tumor cells to contribute towards the oncolytic activity^33^. Besides that, improved oncolysis through a combination of both direct viral infection (intra-tumoral) and immune mechanisms (IL12) has also been demonstrated^33^. This potentially explains the high viral copy number and viral tropism for tumors. Incidentally, the oncoselectivity of NDV is dependent on various intracellular factors such as the expression of specific proteins as well as the extracellular matrix component of the tissues and is thus cell-dependent^33^. Nevertheless, the anti-viral response *in vivo* allows efficient NDV viral clearance, which is essential because the viral vector will no longer be needed after the induction of anti-tumor effects via immunostimulatory cytokine expression^34,35^. Thus the study highlights the ability of rAF-IL12 as an efficient and safe candidate for breast cancer therapy. In the future, the editing of F gene of rAF-IL12 for clinical use will be considered. Hence, further studies should focus on the determination of the apoptotic and anti-metastatic effects of rAF-IL12 against human breast cancer cells to further substantiate the potential of rAF-IL12 as an oncovirotherapeutic.

## Materials and Method

### Plasmids, cells and viruses

The plasmid prAF_poltv5 was obtained from Kavitha Muralitharan (Institute of Bioscience, Universiti Putra Malaysia) and served as the backbone for the modification of the NDV genome and plasmid pUNO1-hIL12 (p40p35) containing IL-12 gene was purchased from Invivogen, USA. The cell lines BHK-21 (ATCC-CCL-10), DF-1 (ATCC-CRL-12203), MCF-7 (ATCC-HTB-22), MDA-MB231 (ATCC-HTB-26), MCF-10A (ATCC-CRL-10317) and 4T1 (CRL-2539) were obtained from the ATCC collection (ATCC, USA). BHK-21 cells were maintained in Glasgow Minimum Essential Media (GMEM) supplemented with 10% Newborn Calf Serum (NCS), 1% tryptose phosphate broth, 1% MEM amino acids and 1% penicillin-streptomycin. Geneticin (G418) was added to the cell culture for selection of cells expressing T7 before transfection (1). MCF-7 and 4T1 cells were maintained in RPMI (Roswell Park Memorial Institute), while DF-1 and MDA-MB231 cells were maintained in DMEM (Dulbecco’s Modified Eagle Medium). All media were supplemented with 10% fetal bovine serum and 1% penicillin-streptomycin. MCF-10A cells on the other hand, were maintained in DMEM F12 media supplemented with hydrocortisone (0.5 μg/mL), insulin (10 μg/mL), hEGF (20 351 ng/mL) and 10% (v/v) FBS (PAA, Austria). All the cells were kept in a 37 °C humidified incubator equipped with 5% CO2. The parental NDV strain, AF2240, a Malaysian strain with a HA unit of 2^10^ was used as a positive control throughout this study. Viruses were purified by 30–60% (w/v) sucrose gradient centrifugation at 275 000 × g for 4 h at 4 °C, and stored in TE buffer (10 mM Tris-Cl pH 8.0, 1 mM EDTA, pH 8.0).

### Construction and rescue of the recombinant virus

The IL-12 gene was amplified using a set of flanking primers include *Nhe1* restriction site, GS, IS, 3′ untranslated region, optimal Kozak sequence, GE and 5′ UTR of IL12 gene. The IL12 gene was amplified from plasmid pUNO1-hIL12 (p40p35). The primer sequences are listed out in Table 3. A restriction site for *Nhe1* was added in the forward primer (also in reverse primer) by mutating the sequences in the 5′ untranslated region of the M gene (5′ untranslated region of IL-12 gene) for cloning purposes. The design for the insertion of transgene is illustrated in Fig. S3. To insert the *Nhe1* restriction site between the M and F genes, two sets of primers were used in overlap extension PCR (OE-PCR). The M/F fragment was amplified from the plasmid prAF_poltv5.

**Table 3:** Sequence of the primers used in the construction of rAF-IL12.

Primer	Sequence
Forward primer	5′GCGCGCTAGCTTACAGTTAGTTCACCTGTTTATCTAGTTAGAAAAAACACGGGTAGAAGAGTTCGGATCCCGATTGGCACATTCAAGGCGCAATACCACCATGTGTCACCAGCAGTTGGTCATCTC 3′
Reverse primer	5′AAGCTAGCCTATTAGGAAGCATTCAGATAGCTCATCACTCTATCAATAGTC 3′
F 1 Forward Primer	ACCAGTACACACCCCTTCCGAGTTA
F 1 Reverse Primer	ATCTGTCTTGCTAGCTTACAGTTAGTTCACCTGTTTATCT
F 2 Forward Primer	CAACACTATATAATGATCTGTCTTGCTAGCTTACAGTTAGTTCA
F 2 Reverse Primer	AATAAGCAATGCCCTGGACAAGTTATCGG

^*^Abbreviation: F = Fragment.

The BHK-21 cells were maintained in 6-well plates prior to transfection until 60–80% confluency. About 1 µg of full-length rAF-IL12 plasmid, 0.4 µg of pCI-neo-NP, 0.2 µg of pCI-neo-P and 0.2 µg of pCI-neo-L helper plasmids were transfected into the BHK-21 cell line using Lipofectamine™ 3000^27,36,37^. The culture supernatant was harvested at 24 hours post transfection and inoculated into 9-day old specific pathogen free (SPF) embryonated chicken eggs.

### Propagation of rAF-IL12

The rAF-IL12 was propagated in allantoic fluid of 9–11 day-old SPF embryonated chicken eggs at 37 °C for 3–4 days. The allantoic fluid was harvested and the replication of virus was confirmed by the HA assay using 1% chicken red blood cells^38^. The obtained virus was then diluted 10^5^ fold for inoculation into new eggs. The propagation was conducted up to passage 10. DF-1 cells on 6-well plates were infected with serially diluted virus and incubated under a 1% methylcellulose overlay at 37 °C for approximately 3–6 days. The plaque size of each variant was obtained by calculating the mean area of 10 plaques and expressed as a value relative to that of the strain rAF-IL12. Experiments were conducted with an MOI of 0.01 for both NDV AF2240 and rAF-IL12.

### Mean death time

To determine the pathogenicity of rAF-IL12 at passage 1, 5 and 10 as well as the parental virus NDV AF2240 in embryonated chicken eggs, MDT was determined according to the OIE manual^39^. Briefly, five 10-day-old embryonated chicken eggs were infected with serial 10-fold dilutions of viruses. The eggs were incubated at 37 °C and monitored twice daily for 7 days. The time to kill the embryos was recorded. The highest dilution that killed all the embryos was determined to be the minimum lethal dose. The MDT was calculated as the mean time for the minimum lethal dose to kill the embryos. The MDT has been used to classify NDV strains into the following groups: velogenic (taking under 60 hours to kill); mesogenic (taking between 60 and 90 hours to kill); and lentogenic (taking more than 90 hours to kill).

### Intracerebral pathogenicity index

The ICPI was performed according to the OIE manual^39^ whereby ten 1-day old chicks were inoculated with 50 µl of infected allantoic fluid with an HA titer of more than 16 HA units/50 µl and diluted 10-fold in PBS. The birds were kept under observation for 8 days and examined daily. The virus isolates that scored an ICPI <0.7 were declared as lentogenic and those with an ICPI  1.5 were considered as velogenic strains of NDV. The NDV strains with intermediate ICPI values were designated as mesogenic. All experiments involving experimental animals were approved by the committee of IACUC, Universiti Putra Malaysia (protocol number R062/2016) and conducted following the guidelines.

### 3-(4, 5-dimethylthiazol-2-yl)-2, 5-diphenyl tetrazolium bromide (MTT) *in vitro* cytotoxic assay

The MTT assay was conducted in accordance to Mosmann^40^ with slight modifications. The cells were seeded in a 96-well plate at a concentration of 0.8 × 10^5^ cells/well and incubated in a 37 °C CO_2_ incubator overnight. The next day, rAF-IL12 at passage 1, 5 and 10 or the parental virus NDV AF2240 was added to the wells at the highest concentration according to their HA unit. The cell viability was measured at 24, 48 and 72 hours post-treatment. Twenty microliter per well of MTT solution (Calbiochem, USA) was added, followed by 4 h incubation at 37 °C in a 5% CO_2_ atmosphere. Then, the supernatants were removed from the wells and 100 μL/well of dimethyl sulfoxide (DMSO) (Fisher, USA) was added to solubilize the formazan. The absorbance at 570 nm was measured using an ELISA microplate reader (Biotech Instruments, USA). Triplicates were carried out for each experiment. The following formula was used to determine the percentage of viable cells:

$${\rm{Percentage}}\,{\rm{of}}\,{\rm{Cell}}\,{\rm{Viability}}=[{\rm{OD}}\,{\rm{Sample}}\,{\rm{at}}\,570{\rm{nm}}/{\rm{OD}}\,{\rm{control}}\,{\rm{at}}\,570{\rm{nm}}]\times 100 \% $$

### Enzyme-linked immunosorbent assay

IL12 concentration was quantified using the DuoSet ELISA Human IL12 p70 kit (R&D systems, Inc., USA) based on suppliers protocol. Upon viral treatment of the cells, the supernatant was collected and loaded into an antibody pre-coated microplate for 2 hours. The plate was washed with a wash buffer and the TMB substrate solution was added in the wells. After adding the stop solution, the absorbance was read on a μ-Quant ELISA Reader (Bio-Tek Instruments, USA) using 450 nm wavelength and 570 nm reference wavelengths.

### *In vivo* cytotoxicity evaluation of rAF-IL12

A group of 5–6 week old female Balb/c mice obtained from Universiti Putra Malaysia’s Animal House were used in this study. The mice were kept under standard conditions at 25 ± 2 °C on a regular light-dark cycle and provided with standard diet pellets and tap water. The mice were divided into 9 mice per group whereby all experiments were conducted according to the Universiti Putra Malaysia’s ethical guidelines for the care of lab animals (UPM/IACUC/AUP-R061/2017).

Untreated and rAF-IL12-treated mice were injected with 1 × 10^5^ cells per mice of the 4T1 cell line subcutaneously. Once the tumor developed after a period of 2 weeks measuring 1 cm, the mice were treated accordingly; untreated (100 µl of PBS), rAF-IL12 (100 µl of 2^7^ HA unit virus) twice a week at the intra-tumoral site. The dose was selected based on a study by Conrad *et al*.^38^. Tumors were measured using vernier calipers twice a week^41^ and the tumor volume was calculated using the following formula: tumor volume (mm^3^) = [(width)^2^ × length]/2. The mice were sacrificed by (exsanguination under) ketamine-xylazine anaesthesia (200 mg ketamine and 20 mg xylazine per kg body weight) at day 28. The sections of organs and tumors were placed in 10% formalin for further histological analysis.

### Viral quantitative reverse by RT-qCR

Total RNA was extracted from the organs and tumors by mechanical disruption and using a Qiagen RNAeasy Mini Kit (Qiagen, USA). The RNA was subjected to reverse transcriptase PCR using iScript cDNA Kit (Biorad, USA) (4 µl of 5× iScript Reverse Transcription Supemix, 6 µl of RNA template and 10 µl of nuclease-free water) incubated in a thermal cycler for 5 min at 25 °C, 30 min at 42 °C and 5 min at 85 °C with the reaction component. The synthesized cDNA was subjected to Taqman real time PCR assay using a reverse primer 5′ CGCTGTTGCAACCCCAAG 3′; forward primer 5′ TCCGCAAGATCCAAGGGTCT 3′ and probe 5′ (FAM)-AAGCGTTTCTGTCTCCTTCCTCCA-(BHQ) 3′ with the annealing temperature set at 58 °C using iQ Supermix (Biorad, USA). The cycling program was set up with 95 °C for 5 min followed by 39 cycles of 95 °C for 10 s, 56 °C for 30 s and 72 °C for 20 s.

### Histopathology staining

The sections of organs and tumor immersed in 10% formalin were paraffin-embedded, stained with haematoxylin and eosin and viewed under a bright-field inverted microscope (Nikon, Japan). Histological architecture and mitotic cells between groups were compared.

### Statistical analysis

The results were analyzed using SPSS Statistics 17 software. The results were expressed as mean ± standard error (SE). Statistically significant differences between the means were determined by One-Way ANOVA followed by Duncan post hoc test. Differences were considered significant when the p ≤ 0.05.

## Supplementary information

Supplementary data

## Data Availability

The authors declare that all the data in this manuscript are available.
