# Supplementary material for: Evaluation of a Recombinant Newcastle Disease Virus Expressing Human IL12 against Human Breast Cancer
Source: Sci Rep. 2019 Sep 30;9:13999. doi: 10.1038/s41598-019-50222-z (PMC6768883; doi:10.1038/s41598-019-50222-z)
Supplement: Supplementary file 1 — Supplementary data [file 41598_2019_50222_MOESM1_ESM.docx]

**Evaluation of a Recombinant Newcastle Disease Virus Expressing Human IL12 against Human Breast Cancer**

**Zahiah Mohamed Amin^1^, Muhammad Alhapis Che Ani^2^, Sheau Wei Tan^1^, Swee Keong Yeap^3^, Noorjahan Banu Alitheen^1,2^, Syed Umar Faruq Syed Najmuddin^1^, Jeevanathan Kalyanasundram^2^, Soon Choy Chan^4^, Abhi Veerakumarasivam^5,6^, Suet Lin Chia^1,2^ &** [**Khatijah Yusoff**](https://www.researchsea.com/html/experts.php/eid/363/research/khatijah_yusoff__dsis__fasc.html)**^2,6^**^*^

^1^ *Institute of Bioscience, Universiti Putra Malaysia, 43400 Serdang, Selangor, Malaysia*

^2^ *Faculty of Biotechnology and Biomolecular Sciences, Universiti Putra Malaysia, 43400 Serdang, Selangor, Malaysia*

^3^ *Xiamen University Malaysia, Jalan Sunsuria, Bandar Sunsuria, 43900 Sepang, Selangor Darul Ehsan, Malaysia*

^4^ *Perdana University, Block B and D1, MAEPS Building, MARDI Complex Jalan MAEPS Perdana, 43400 Seri Kembangan, Selangor Darul Ehsan, Malaysia Ehsan, Malaysia*

^5^ *Department of Biological Sciences, School of Science and Technology*, *Sunway University, 5, Jalan Universiti, Bandar Sunway, 47500 Subang Jaya, Selangor Darul*

*^6^ Malaysian Genome Institute, Jalan Bangi, 43000 Kajang, Selangor, Malaysia*

Correspondence should be addressed to [Khatijah Yusoff](https://www.researchsea.com/html/experts.php/eid/363/research/khatijah_yusoff__dsis__fasc.html); [kyusoff@upm.edu.my](mailto:kyusoff@upm.edu.my)

**Supplementary information**

0.5 kb

1 kb

2 kb

3 kb

**Fig. S1** PCR was carried out amplify intergenic region of each gene in plasmid rAF-IL12 using specific primers. NP/P region indicates region between NP and P gene, P/M indicates region between P and M gene, M/F indicates region between M and F gene, F/HN indicates region between F and HN gene and HN/L indicates region between HN and L gene. The dashed lines separate samples unrelated to this study.

Ladder NP/P P/M M/F F/HN HN/L

**Fig. S2** PCR was carried out to amplify the intergenic region of each gene in the rAF-IL12 plasmid. Lane 1&2: NP/P region; Lane 3/4: P/M region; Lane 5/6: M/F region; Lane 7/8: F/HN region and; Lane 9/10: HN/L region.
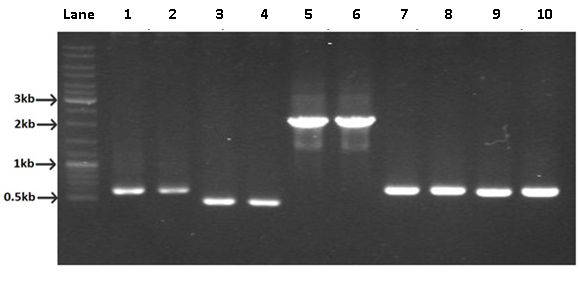


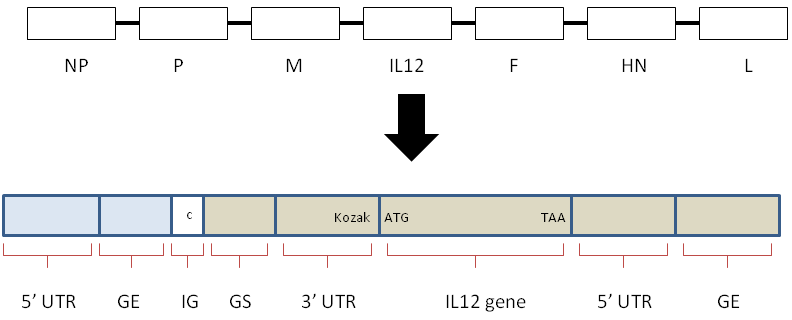


**Fig. S3** Design construction of rAF-IL12. The insertion of transgene, Kozak sequence was included preceding the start codon of IL-12 gene in the 3’ UTR region. Upstream of IL12 gene, IG, GS and 3’ UTR was inserted allowing the replication and transcription of the transgene.

UTR: Untranslated region; IG: Intergenic sequence; GS: Gene start; GE: Gene end.
